# Strategies to promote evidence use for health programme improvement: learning from the experiences of embedded implementation research teams in Latin America and the Caribbean

**DOI:** 10.1186/s12961-022-00834-1

**Published:** 2022-04-07

**Authors:** N. Ilona Varallyay, Caitlin Kennedy, Sara C. Bennett, David H. Peters

**Affiliations:** 1 Health Systems Program, Department of International Health, Johns Hopkins School of Public Health, 615 N Wolfe St, Baltimore, MD 21205 United States of America; 2 Social and Behavioral Interventions Program, Department of International Health, Johns Hopkins School of Public Health, 615 N Wolfe St, Baltimore, MD 21205 United States of America; 3 Department of International Health, Johns Hopkins School of Public Health, 615 N Wolfe St, Baltimore, MD 21205 United States of America

**Keywords:** Implementation research, Embedded research, Evidence coproduction, Evidence-informed decision-making, Knowledge translation, Health policy and systems research, Low- and middle-income countries, Latin America and the Caribbean

## Abstract

**Background:**

To achieve global health targets, innovative approaches are needed to strengthen the implementation of efficacious interventions. New approaches in implementation research that bring together health system decision-makers alongside researchers to collaboratively design, produce and apply research evidence are gaining traction. Embedded implementation research (EIR) approaches led by decision-maker principal investigators (DM PIs) appear promising in this regard. Our aim is to describe the strategies study teams employ in the post-research phase of EIR to promote evidence-informed programme or policy improvement.

**Methods:**

We conducted a prospective, comparative case study of an EIR initiative in Bolivia, Colombia and Dominican Republic. Guided by a conceptual framework on EIR, we used semi-structured key informant interviews (*n* = 51) and document reviews (*n* = 20) to examine three decision-maker-led study teams (“cases”). Focusing on three processes (communication/dissemination, stakeholder engagement with evidence, integrating evidence in decision-making) and the main outcome (enacting improvements), we used thematic analysis to identify associated strategies and enabling or hindering factors.

**Results:**

Across cases, we observed diverse strategies, shaped substantially by whether the DM PI was positioned to lead the response to study findings within their sphere of work. We found two primary change pathways: (1) DM PIs implement remedial measures directly, and (2) DM PIs seek to influence other stakeholders to respond to study findings. Throughout the post-research phase, EIR teams adapted research use strategies based on the evolving context.

**Conclusions:**

EIR led by well-positioned DM PIs can facilitate impactful research translation efforts. We draw lessons around the importance of (1) understanding DM PI positionality, (2) ongoing assessment of the evolving context and stakeholders and (3) iterative adaptation to dynamic, uncertain circumstances. Findings may guide EIR practitioners in planning and conducting fit-for-purpose and context-sensitive strategies to advance the use of evidence for programme improvement.

**Supplementary Information:**

The online version contains supplementary material available at 10.1186/s12961-022-00834-1.

## Introduction

### Background

Globally, many scientifically proven public health interventions fail to attain desired health outcomes [1]. To address such challenges, we must not only bridge the gap between science and practice, but also invest in research on the implementation of these interventions in different contexts [2]. This is particularly true for low- and middle-income country (LMIC) settings, where resource scarcity demands effective and efficient implementation of critical health programmes and policies to achieve global health targets such as the Sustainable Development Goals [3, 4]. New approaches are needed to promote the use of locally produced research evidence to inform health programme or policy improvement.

Embedded approaches that place health system decision-makers at the helm of implementation research (IR) are emerging as innovative and potentially impactful in this regard. Health system decision-makers include policymakers, health programme managers or directors, regional health department officers, implementers and even some frontline health workers. Embedded implementation research (EIR) aims to advance evidence-informed decision-making (EIDM) by situating decision-makers in a lead role throughout research production as well as the application of evidence to practice. Decision-maker ownership over the research endeavour can be fostered by promoting their direct participation in determining the need for research; identifying a priority programme/policy problem to be examined; formulating research questions to align with local information needs for decision-making; engaging pivotal decision-makers and facilitating access to resources and decision-making spaces; grounding the interpretation of findings within local realities to develop feasible, context-sensitive solutions; informing and implementing a research dissemination plan; and taking direct action informed by the evidence to improve programmes/policies.

The active engagement of decision-makers and other “knowledge users” in research processes has long been recognized as a way to enhance the application of research evidence in health programme or policy decision-making processes [5–7], highlighting the value of “interaction” or “linkage and exchange” [8] between researchers and decision-makers. Such a role for decision-makers is particularly well suited to IR, which studies the delivery of evidence-informed health services, programmes and policies in different contexts and scales “to improve [their] effectiveness, quality, efficiency and equity” [9]. With a view to optimize delivery of critical scientifically proven health interventions in local settings, IR entails systematic examination of the various factors that may affect implementation—local context, health system capacity, organizational culture, provider behaviour, etc.—in addition to the actual processes and outcomes of implementation [10]. As such, health system decision-makers are well positioned to provide key insights into both research processes—thereby augmenting the relevance, timeliness and applicability of research for problem-solving [6, 11–13]—and processes to ensure research utilization for programme improvement [14].

The EIR initiative of interest in this paper, Improving Program Implementation through Embedded Research (iPIER) [15, 16], was supported in Latin America and the Caribbean (LAC) by the WHO Alliance for Health Policy and Systems Research (the Alliance) and the Pan American Health Organization (PAHO). A previous retrospective analysis of barriers and facilitators to EIR suggests this approach has the potential to enhance use of research evidence by decision-makers for programme/policy improvement [17]. However, there is limited understanding of *how* this engagement of decision-makers and their “interaction” with researchers influences research utilization in the context of EIR approaches. If decision-maker-led IR approaches are effective, then it is important to understand the specific pathways through which EIR teams contribute to “research use” in its various forms—i.e. direct application in decisions about implementation (instrumental) or indirect processes that shift stakeholder understanding and attitudes toward the issue (conceptual) [18, 19]. Advancing EIR practice requires a deeper understanding of the *how* and *why* of the embedded approach—particularly in linking key processes and outcomes.

With growing attention to the potential impact of these demand-driven embedded research approaches [12], calls have been made in the literature for their systematic evaluation [21]. The study conducted here is the first prospective evaluation to systematically examine not only the research endeavour and effects, but also post-research processes and decision-making outcomes. Our interest was to examine both the strategies EIR teams employ to promote research uptake in the later-stage processes of EIDM (“post-research processes” hereafter) as well as the factors that influence the effectiveness of these strategies. Strategies are broadly defined as “observable actions designed to achieve an outcome” [22]. The post-research processes include (1) communicating and disseminating the research to relevant stakeholders; (2) engaging stakeholders with the research evidence to problem-solve and jointly identify remedial action; and (3) integrating evidence in decision-making (negotiations, resource allocation, approvals)—all of which are expected to contribute to enacting changes to programmes or policies, either promoting health system readiness for change, or directly introducing implementation improvements [23]. While these processes focus on the final stages of the research, we acknowledge that study teams can begin setting these up from the outset (and over the course) of a study. We deliberately address these later-stage processes as a previous study focused on the research phase, examining how the features of this specific EIR model were operationalized and how they shaped both research process and outcomes [24].

The qualitative comparative case study presented here aims to provide practical learning for EIR implementers about the “good practices” they can employ to advance evidence-informed action. We do so by addressing two research questions:

- How (through what strategies) did EIR teams aim to promote the use of their research findings for programme/policy improvement (i.e. research utilization) and with what effect?
- What key factors or conditions influenced (enabled/hindered) the contribution of these strategies to the overall goal of evidence-informed programme/policy improvements in different contexts?

## Methods

We conducted a prospective comparative case study of EIR projects implemented between 2016 and 2017 using qualitative methods. The study was guided by our working conceptual framework [23] and focused on the later phases of EIDM.

### Study population

The iPIER initiative, funded jointly by the Alliance and PAHO, promotes an innovative approach to EIR, wherein health programme and policy decision-makers are supported to conduct research intended to improve their programmes/policies. This model of EIR advances four key features: (1) the central role of health system decision-makers in the conduct and use of research, (2) a research focus on issues of programme/policy implementation, (3) collaborative partnerships between researchers and health system decision-makers and (4) integration of research and programme/policy processes [23]. The utilization of research evidence to inform programme changes was an explicit programme objective.

Between 2014 and 2017, iPIER disbursed grants to 10 countries (totalling 19 projects); this is the first initiative in the LAC region to implement this model of EIR. The 2016–2017 cohort funded seven projects (approximately US$ 30 000–35 000 each), supported by technical assistance (TA) from researchers at the National Institute for Public Health of Mexico for the duration of the research phase. No support was provided in the post-research phase, which in part motivated our focus on this phase. These IR studies aimed to understand the root causes of an implementation problem to devise solutions.

### Case selection

We defined the “case” as an IR project led by health system decision-makers with the aim of improving the implementation of an existing health policy or programme. Cases were purposively selected based on characteristics expected to influence the creation, uptake and application of evidence: decision-maker self-designated as principal investigator; decision-maker initiated response to call for IR proposals; decision-maker conceptualized research topic/questions; decision-maker position/role within targeted programme; and stage reached at grant completion. Three cases were selected that either (1) exhibited the key EIR features postulated as contributing to success in our conceptual framework (“most likely” cases) or (2) lacked one or more of the critical features, with the expectation that these would not succeed (“least likely” cases) [25, 26]. This approach was driven by our goal of analytical generalizability [27] wherein the empirical findings from our cases can be used to generalize to the theory of the role of EIR in EIDM and thereby generate practical lessons for consideration by future EIR initiatives [28].

Colombia and the Dominican Republic (DR) were selected as “most likely” cases and Bolivia as a “least likely” case. Table 1 provides a summary of the public health intervention, research objective, and methods used by each case.

**Table 1:** Summary of research projects for each case

	Bolivia	Colombia	DR
	Expected “least likely” case	Expected “most likely” case	Expected “most likely” case
Targeted programme	*Chispitas* micronutrient supplement (one intervention within the wider national micronutrient strategy)	*Por Ti Mujer* (PTM) cervical cancer programme, focusing on the screening component	National Family Planning Programme (FPP) within the MoPH Sexual and Reproductive Health Programme, focusing on male contraception component (vasectomy, condoms)
Research objective	To understand the barriers and facilitators related to consumption and adherence of the *Chispitas* in children 6–23 months	To identify the strategies related to the access and quality of care in the public health services of Cali that may affect coverage of cervical cancer screening services	To identify mechanisms in the implementation of the FPP that facilitate or constitute barriers to the effective integration of men as a beneficiary population
Methods	Qualitative (semi-structured interviews, focus groups and direct observations)	Qualitative (semi-structured interviews, direct observations, focus groups, and document review)	Mixed methods (1) Review of the literature (2) Qualitative: semi-structured interviews focus groups (3) Quantitative: survey of healthcare providers

### Data collection and management

Three rounds of data collection were conducted by the lead author between August 2018 and July 2019 (11 to 24 months post-research completion). Data was obtained from semi-structured key informant interviews, document review, and researcher memos [29]. Key informant interview respondents included EIR study team members (co-investigators) and other stakeholders (including potential knowledge users). Interview guides were developed to incorporate key elements of the conceptual framework (with adaptations by respondent category and stage of EIR) and piloted with one of the decision-maker PIs with research experience (Bolivia) to streamline questions, clarify wording and identify adaptations (e.g. probes) necessary by case; a follow-up interview was subsequently conducted with this respondent to further refine the guide. We sought to elicit factual information about processes and outcomes as well as perceptions about the EIR experience. All interviews were conducted in Spanish, audio recorded with consent and transcribed. Memoing was conducted immediately after interviews and also intermittently throughout data collection to capture emerging methodological and analytical reflections. In total, 51 initial and follow-up interviews were conducted with 37 respondents (Table 2). The first and third rounds of data collection focused on interviews with EIR co-investigators, conducted using Skype; prior contact with these respondents in another study [23] facilitated this modality. Field visits were conducted to each country in the second round, allowing for in-person interviews with system stakeholders external to the study team. Interviews lasted between 35 and 85 min; most lasted approximately an hour. Documentary evidence (*n* = 20), such as media articles, annual plan budgets, research-based infographics, and final reports, were reviewed to confirm statements about key achievements reported. MAXQDA (version 18.1.1) data management software was used to facilitate data coding, organization and retrieval.

**Table 2:** Number of interview respondents by category per country

Respondent category	Bolivia	Colombia	DR
Research team			
Decision-maker co-PI	3^a^	2^a^	2^a^
Researcher co-PI	None	None	2^a^
External health system stakeholders			
Public sector (e.g. Ministry of Health/government)	7	11	5
Other^b^	n/a	1	4

^a^Indicates that at least one follow-up interview was conducted; note that in round three of data collection, it was not possible to interview the two co-investigators on the Bolivia team nor the two researchers on the DR team

^b^“Other” includes actors in the health system that are not formally affiliated with the national Ministry of Health, such as private health insurance companies, professional associations, nongovernmental organizations; composition varied according to context

### Data analysis

The analytical phase was an iterative process, woven intermittently into data collection and led by the lead author. The initial phase focused on descriptive analysis with a view to develop detailed case study descriptions. This involved reviewing transcripts to become familiar with the data and coding transcripts using an a priori coding structure based on our working conceptual framework [23]. As new codes emerged, these were incorporated into the codebook, and transcripts were recoded as needed. Throughout the coding process, the lead author wrote memos documenting issues with coding as well as preliminary observations and interpretations of salient codes. Once each data collection round was completed, coded segments were summarized and charted to develop summary matrices organized around the conceptual framework process steps for each case, capturing supporting raw data (quotes) from different sources and respondent categories [30, 31]. This allowed for triangulation by both data source and across interviewee perspectives, which was useful in corroborating reported outcomes.

#### Within-case analysis

In the subsequent within-case explanatory analysis, the lead author used deductive thematic analysis to interrogate the case-specific summary matrices. This focused on identifying linkages between the following analytical domains: (1) the strategies implemented by the EIR teams in the post-research phase to advance the ultimate goal of EIDM, (2) the main achievements these strategies contributed to—which reflect the effectiveness of strategies, and (3) the key factors that were reported or observed to influence the overall processes of change—including, but not limited to “context”. We summarized results of the thematic analysis [32] in tables created for each of these three analytical components, which were subsequently used to guide the cross-case analysis. The analytical domains “EIR strategies” and “achievements” were organized around the key process components; the domain “influencing factors” was organized according to predetermined constructs, in addition to a few emergent concepts from the thematic analysis. Through this process, we reached saturation regarding our research questions about EIR strategies. To ensure the integrity of the analysis, the lead author periodically reverted back to the coded segments (raw data) to confirm that the evolving interpretations were coherent and well grounded in the data [33].

#### Cross-case analysis

The cross-case analysis focused on comparing the strategies employed and disentangling the most prominent factors that shaped the progress of each team in advancing the use of research. We examined overarching pathway(s) of change, identifying commonalities/distinctions and highlighting the relevance of different EIR strategies. Based on this analysis, we distilled practical lessons to guide EIR teams in planning and conducting appropriate post-research strategies.

### Ethical approval

A non-human subjects research exemption was received for this study from both the PAHO Ethical Review Board (PAHO-2018-06-0042) and the Johns Hopkins School of Public Health Institutional Review Board (dated 23 March 2018).

## Results

### Case study descriptions

#### Case 1: Incorporating men into the family planning programme in DR (“most likely” case)

The DR case demonstrated several strategies relevant for EIR aimed at incremental policy-level change. The co-principal investigator (Co-PI 1) with a decision-making role in the family planning (FP) programme pursued research use strategies by leveraging opportunities in her routine professional functions. For example, in annual programme planning and budgeting processes, she introduced measures to enhance system readiness for change, such as health provider trainings on male contraception; in policy (re)formulation processes, she facilitated the integration of male contraception into national FP service delivery norms and guidelines. At the time our study concluded, however, these approved normative documents had not yet been formally “launched” by higher-level ministry officials due to inexplicable delays; study team members expressed concerns that the political context (pre-election) impeded any action that might significantly alter the status quo. This essentially precluded implementation of measures to address study findings, despite being budgeted in the annual plan (e.g. publication and distribution of new protocols/guidelines and related health provider trainings). With regard to changes in attitudes about the issue, the Co-PI 1 reported reflective thinking about the evidence as a strategy that allowed her to understand and adopt previously proposed (but resisted) changes to specific programme arrangements, such as transferring responsibilities from the Ministry of Public Health (MoPH) to the newly formed entity charged with health service delivery oversight—the National Health Services (SNS, acronym in Spanish).

In terms of stimulating action among other system stakeholders, the EIR team pursued a combination of strategies. Co-PI 1 facilitated direct access to senior leadership within the relevant MoPH department. The study team organized a formal presentation of findings to high-level stakeholders, including the Minister of Health. To ensure that desired policy development was acceptable to senior MoPH authorities, Co-PI 1 conducted periodic informal meetings to discuss implications of research findings for action. These deliberate dissemination and stakeholder engagement efforts were reported to have paved the way for use of findings by a few pivotal stakeholders closely tied to the FP programme. Other stakeholders were more difficult to mobilize to action. Critically, stakeholders from the SNS—those most directly implicated by findings—were focused on setting up key functions as a new government body and unable to pursue the cause of male contraception, despite reported sympathy with the overall study goal. As one SNS official stated, “*This [EIR] study … provides credible, reliable findings … and must form part of the documentary portfolio that will allow the MoPH to continue promoting and raising the visibility of male contraception as a gender issue*.” To elicit support from high-level officials, the study team decided to shift the framing of key findings to align with political priorities in the health sector (e.g. reduction of maternal mortality). With regard to dissemination of results, the team commissioned a professionally designed infographics brochure summarizing key findings for mass distribution. Considering the inter-sectoral nature of the issue, the team organized a deliberative action-planning workshop engaging diverse stakeholders to problem-solve and develop remedial measures based on study findings. While the exercise itself was constructive—producing a detailed multi-sectoral action plan—challenges arose in its execution due to the absence of an adequate mechanism holding all parties accountable.

A few features of the wider context appear to have hindered aspects of the overall change process. For example, the pre-election political context as well as the predominantly conservative culture and associated assumptions about men’s role in health were reported to foment system-wide resistance to change. Even in favourable circumstances, the nature of the desired change—i.e. the introduction of new services (vasectomy) across lower-level health facilities—would require time-intensive installation and roll-out measures. Furthermore, the creation of the new health sector operational arm (SNS) occurred over the course of the iPIER study, which created two key challenges for the EIR team: (1) cultivating relationships with SNS early on and (2) obtaining their buy-in for male contraception in the context of other priorities.

In exploring potential alternative explanations for the advances observed—i.e. changes to service guidelines, inclusion of system readiness activities in the annual budget—no other factors emerged that could have contributed more significantly than the EIR endeavour. There was some evidence of increased interest sector-wide in the role of men in health during the study time frame. However, most respondents expressed a perception that the “sound and credible” scientific evidence from the EIR presented a compelling case for change, ultimately propelling some stakeholders to act. A senior Ministry official noted: “*For my division to be able to raise attention to the issue within the entire team, for me that was the main action. And that when talking about developing the new departmental annual plan, everyone considered [the role of] men as beneficiaries. So, for me, that was a fundamental step. I believe that this will be the greatest contribution that the research had on my team had—being able to awaken actions on this issue*.”

#### Case 2: Addressing access and quality to improve coverage of the cervical cancer screening programme in Colombia (“most likely” case)

In Colombia, the strategies required to catalyse evidence-informed change were relatively straightforward, and the decision-maker principal investigator (DM PI) was able to respond directly to study findings within the health network she managed. Early on, the DM PI took a clear decision to focus deliberately on applying findings within her direct locus of authority. The DM PI reported this was based on recognition of her lack of authority to oversee (or enforce) remedial measures by other stakeholders (i.e. other health networks, health insurance companies, Health Secretariat): “*The idea was never to follow up with all the [other] actors. It was simply to share the information and let them decide what they decide with their own criteria. […] We do not have authority over the other networks […] neither can we follow up, because we cannot be their bosses. They can’t feel like we’re evaluating [them]*.” The DM PI explicitly focused on “micro” changes at the operational/administrative level within her network, where she was directly able to supervise implementation (e.g. changing operating hours to accommodate service users’ needs; negotiating arrangements with health insurers to shorten patients’ delays in accessing services; simplification of registration and billing processes to reduce wait times; expanding health promotion teams to address lack of medical staff). Many of these were implemented in real time, as the data collection and analysis were being finalized. The minor resource implications of these measures obviated formal, higher-level approval processes and facilitated swift action. We observed that existing service quality improvement processes facilitated introduction of these changes as well as their monitoring; this helped identify the effects of some measures and course correct as needed. While a formal public presentation on findings was made, more intensive “dissemination” and “engagement” processes targeting external stakeholders were largely circumvented; within the health network, findings were incorporated into existing staff communication channels. Other stakeholder engagement strategies, such as deliberative consultations or joint problem-solving, were not deemed necessary—key informant interviews from the IR study already elicited stakeholders’ views on remedial measures.

In combination, the programme changes implemented were reported to have contributed to improving health network performance and service quality—which was reflected in monthly patient exit interviews. The DM PI did note, however, that desired changes in coverage of screening services—the main “implementation outcome” [34]—were not achieved. Wider system-level factors beyond the DM PI’s control, including the inadequate health promotion activities at the municipal level by public health insurance entities as well as cultural barriers among women, were mentioned as contributing factors. The DM PI commented that without this research grant, she would have nonetheless pursued strategies to improve coverage. Whether other efforts would have led to the same discoveries in the same rapid time frame is unknown, particularly given the discrepancies she noted between study findings and information about programme implementation previously reported by her staff.

While the iPIER study certainly contributed to catalysing improvements, several external stakeholders mentioned these changes could not be attributed entirely to the iPIER study, as other parallel initiatives (e.g. the Municipal Decennial Plan for Cancer) also created supporting conditions. Furthermore, the competitive environment in the local health market likely incentivized other health network managers who reported implementing corrective measures (arising from the study) in their own jurisdictions—despite not having been actively engaged by the study team.

#### Case 3: Understanding barriers and facilitators to *Chispitas* anaemia micronutrient supplementation in Bolivia (“least likely” case)

In Bolivia, the DM PI proactively pursued several strategies to promote the use of research on micronutrient supplementation by suitable stakeholders. However, direct remedial action was not an option within her own sphere of decision-making at the municipal hospital, given that this was not the locus of needed change. She led intensive results dissemination efforts with key stakeholders at various levels of the system (from community health programmes to local health workers/networks to the Ministry of Health [MOH]); this process involved gauging responsiveness of actors and reassessing which audiences to approach. The DM PI also engaged some of these stakeholders in targeted meetings, adapting message framing for each audience. Dissemination also included distribution of a compact disc (CD) with the study report and other implementation resources on *Chispitas* for frontline health workers. The dissemination and engagement efforts, which were carried out in a largely ad hoc manner through one-off activities, triggered several unexpected, reactive measures by frontline health workers (e.g. conducting a local anaemia prevalence study; further dissemination of findings to other frontline staff). While these did not specifically respond to study findings, they nonetheless contributed to raising awareness of the issue. A key challenge to integrating the findings into decision-making was the absence of the *Chispitas* programme structure per se, creating uncertainty about how best to influence local implementation decisions. Insufficient interest in the issue by the local health network coordinator—the stakeholder best placed to coordinate local level remedial action—further compounded this dilemma. Instead, the DM PI sought to influence provider behaviour through dissemination of findings to the Health Information and Analysis committees (a key facility-level decision-making structure) across the health network.

At the central level, the relevant MOH officials expressed strong interest in and appreciation for the study: “*I have emphasized the importance of the study because it serves as scientific support, as scientific management to take actions—and not just at the level of the Municipality but at the national level, […] because the reality [across the departments] is very similar*.” These officials supported its dissemination (e.g. presenting study findings at a national nutrition conference; reactivating the MOH Anaemia Roundtable as a space for deliberation). They even proposed specific remedial measures; however, it appears these were not acted on. The DM PI mentioned limitations to the role she could play in promoting research use; it was not her place to coordinate stakeholders to devise or implement remedial measures based on findings. No other stakeholder or entity stepped into this role. Instead, she exerted pressure on the Anaemia Roundtable (on which she sat as a technical expert) to reconvene for the purpose of presenting study findings. Ultimately, this key decision-making body appears to have focused selectively on certain findings in revising the anaemia strategy—e.g. proposing (a) an increased *Chispitas* dosage despite findings about the sociocultural barriers to intervention uptake among caregivers and (b) a mass information and education campaign targeting behaviour change among beneficiaries, ignoring evidence about root causes of low adherence unrelated to gaps in caregiver knowledge. While we could not uncover the reasons for this seeming disconnect (neither could the DM PI), ministry respondents expressed clear aversion to modifying the *Chispitas* product; one MOH policymaker said, “*We as the MOH Nutrition Unit have argued that while the [Chispitas] scheme is in place, there will be no changes. It remains valid, and what we have to do is continue to strengthen, we have to comply with the scheme*.” Ultimately, other factors appear to have taken precedence in proposals to reformulate the anaemia policy. DM PI comments regarding the extensive investments in local production of *Chispitas* and the increasing evidence about its ineffectiveness (in contrast to success internationally) suggest possible political motivations for failing to consider changes to the *Chispitas* intervention itself.

In addition to the underlying political interests, other dimensions of the wider context appear to have shaped the outcomes of this case: health system “shocks” that presented competing priorities to high-level decision-makers (e.g. protracted health sector strike; roll-out of universal health coverage plan); the politically fragmented structure of the public governance system across levels, exposing decision-making to internecine party politics and impeding coordinated action; and, not least, the transfer of the two local decision-maker co-investigators early in the research, hindering their continued involvement in post-research activities. Additional information on the study findings and recommendations for each country can be found in Additional File 1.

### Cross-case analysis of strategies in late phases of EIDM

#### Communication and dissemination

Across all cases, study teams actively communicated research findings to initiate processes of change among potential knowledge users; these efforts were primarily driven at the individual (rather than organizational) level by study team members (notably the DM PIs). The importance of effective communication for ensuring research impact has been reported previously [35]. However, in two of our cases, extensive dissemination to other stakeholders was not required to implement remedial measures. Instead, opportunities for direct action by DM PIs were pivotal to advance evidence-informed changes. While all teams expressed the intention of “acting” on the evidence at the outset—an explicit objective of iPIER—the appropriate scope of dissemination strategies depended largely on the nature of the desired change relative to both DM PI level of authority (and sphere of influence) and the breadth of stakeholders implicated in requisite change processes. Several themes related to study teams’ communication and dissemination strategies emerged from our analysis:

- Direct access to key stakeholders: All EIR study teams were able to access the key potential research users directly; none reported challenges with gatekeepers or other intermediaries. This reflects the ability of study team members to draw on their professional networks in disseminating findings. However, these linkages were often insufficient in compelling stakeholders to action—depending on factors outside the study teams’ control (e.g. perceived incentives to act).
- Informal stakeholder analysis: Study team respondents in DR and Bolivia identified a range of stakeholders for dissemination, primarily drawing on their existing knowledge about the context and expectations about how relevant actors might intervene. Notably absent across cases was any mention of structured processes to identify target audiences (e.g. stakeholder analysis, strategic communication plan), which are encouraged in the literature [36–38]. Our interpretation is that the DM co-investigators’ “insider knowledge” about contextual barriers and opportunities was perceived as sufficient in guiding such decisions.
- Aligning message framing to local context: All three teams described deliberate decisions around framing key messages about findings in a way that resonated with top health system priorities (DR), eschewed politicization of research through politically neutral framing for high-level stakeholders (Bolivia), and set a tone to motivate frontline health workers to action (Bolivia and Colombia).
- Unclear utility of knowledge products (study reports, evidence briefs, brochures, etc.): While all teams prepared and disseminated formal study reports to key stakeholders, only two teams developed additional knowledge products intended to help promote wide-scale behaviour change of target audiences (a CD in Bolivia and infographics brochure in DR). Given the timing of their development, we were not able to link the use of these products with the most significant changes observed—challenges other studies have also noted [39]. Colombia did not report developing additional communication tools, which aligns with their deliberately narrow focus within the health network. While all teams mentioned interest in subsequently writing a scientific manuscript on their IR studies, none pursued this as a primary mechanism to influence programme/policy changes.

### Engaging stakeholders with evidence

Stakeholder engagement with evidence differs from “dissemination” as it goes beyond one-way sharing of information and is typically more interactive and purposeful. While stakeholder engagement began in the pre-research and research phases, we observed two primary “modes” of engaging stakeholders once the evidence was produced: targeted one-on-one meetings or discussions with pivotal stakeholders and more structured, deliberative problem-solving processes.

- Targeted meetings with pivotal stakeholders: These meetings were carried out by study team members (mainly DM PIs) to convince diverse stakeholders of the need to act on study findings and devise remedial strategies. In DR, such personal communications aimed to: follow up on commitments expressed during joint action planning; engage actors that did not attend the dissemination meeting; secure technical and financial support from nongovernmental organization for the development of a revised contraceptive protocol; and maintain the MoPH unit leadership abreast of developments. Such engagement was needed to focalize stakeholders’ support for study recommendations. In Bolivia, intensive efforts made by the DM PI to engage a wide range of stakeholders with the evidence had greatest effect with local level providers; at the central level, it proved difficult to move beyond communicating knowledge to exerting influence on stakeholders to act. In Colombia, where such external stakeholder meetings were not reported, it is possible that discussions about study findings were integrated into routine meetings or other informal interactions with internal staff and therefore not reported.
- Deliberative processes: Though multi-stakeholder deliberative processes are promoted as strategies effectively promoting research use through linkage and exchange [8, 40], DR was the only case in which such processes were deemed necessary and feasible by the study team. While these processes were useful in generating multi-sectoral remedial action plans, study team members identified two conditions that ultimately interfered with desired effects: (1) absence of stakeholders with adequate decision-making and resource allocation authority and (2) failure to identify suitable accountability mechanisms to oversee coordinated plan implementation.

These stakeholder engagement activities were intended to foster buy-in towards the study among external stakeholders and motivate them to respond to study recommendations. In contexts where key remedial measures were beyond the reach of DM PIs, these strategies helped elicit concern, or a sense of responsibility, over the issue among other key stakeholders. In the case of DR, buy-in was most evident among the MoPH department stakeholders—who were engaged most regularly by the study team, as they were directly implicated by the study. In Bolivia, local frontline health workers demonstrated the strongest buy-in to the findings, taking actions motivated by self-reported sense of responsibility over gaps identified by the study.

### Integration of evidence in decision-making

Ensuring that research evidence is fed back into decision-making processes is critical in evidence-to-action efforts. In all three cases, study team members brought the evidence into some local decision-making processes or spaces (formal or informal). In both Colombia and DR, this integration occurred directly through the DM PIs as they engaged in their routine work, reflecting their ownership and commitment to ensure findings are used to improve programmes/policies. In DR, Co-PI 1 leveraged her routine responsibilities—e.g. development of the FP annual plan—as opportunities to integrate key findings into policy directives, thereby establishing a path toward system readiness for implementation changes. In Colombia, the DM PI—the primary actor responsible for optimal programme performance—considered the implications of the evidence and directed and oversaw remedial measures. Key findings were also fed into existing quality improvement processes, which supported related decision-making processes. In Bolivia, the DM PI’s routine decision-making sphere at the municipal hospital was not the focus of proposed remedial measures. Instead, she leveraged her membership within the MOH-led Anaemia Roundtable to ensure study findings were reviewed—however, other considerations appear to have driven decisions on the *Chispitas* regimen.

Across all cases, the DM PIs reported the ways in which research evidence was considered and reshaped their (or key stakeholders’) understanding of the problem or the needed solutions. We note that while such conceptual use of research was consequential in some circumstances, it did not always lead to more impactful change, that is, improvements to programme/policy.

### Pathways of change

Two principal change pathways emerged: (1) a direct pathway in which DM PIs themselves are able to implement remedial actions, in some circumstances enabling real-time changes and (2) an indirect pathway in which the DM PI must engage and influence other pivotal system stakeholders to pursue remedial measures—an important distinction [41] that shapes late-stage EIDM strategies. These two approaches are not mutually exclusive; instead, our study indicates that both may be necessary—as transpired in DR—depending on the nature and level of desired change. A central enabling factor for both approaches is the alignment of the DM PI sphere of influence and the locus of change emanating from the research.

## Discussion

This study aimed to elucidate the processes through which EIR teams advanced the use of the research knowledge they had generated for health programme and policy improvement. The lessons that emerged about post-research EIR strategies highlight the importance of three critical areas: understanding DM PI authority and decision-making power, ongoing assessment of the evolving context and relevant stakeholders, and iterative adaptation to dynamic and at times uncertain circumstances—all of which reflect the complexity involved. One dimension of this complexity relates to the influence of starting conditions, such that decisions in the pre-research and research phases have direct consequences on the course of action in the post-research phase. We thus note some considerations from earlier phases that bear directly on the later processes of EIDM. Additional File 2 provides a summary of key practical considerations emanating from this study.

### Understanding DM PI positionality and implications for post-research planning

The rationale behind the EIR model studied here rests on the notion that if the research problem is conceptualized (and the endeavour subsequently led) by suitable programme/policy decision-makers, there is a good chance that at least some responsive measures can be implemented. A previous study on EIR demonstrated the ways DM PI positionality influences the research endeavour, highlighting the importance of DM PI “insider” status, their scope of authority over the programme/policy, and other factors [24].

In this study, we found that while post-research EIR strategies varied considerably across cases, the primary “change agents” [42] were the DM PIs. Our cases have shown the DM PI positionality as an important determinant of the specific strategies needed in the post-research phase to advance the use of evidence for programme improvement. This aligns with prior research demonstrating that studies initiated by “people who were primary decision-makers or held influential positions in the health system” (p. 11) were the most likely to translate research into action [39]. In our study, in cases where the DM PI viewed the application of the research as “part of their normal work” (DM PI respondent), research uptake processes gained greater traction, affirming the underlying rationale of this EIR model. A key enabling factor appears to be the alignment between the positionality of the DM PI and the locus of decision-making implicated by the study findings.

“Who” is involved in driving evidence use is as critical as the processes they implement [43]. In light of this, we see great benefit for EIR teams to reflect explicitly on the implications of the study team’s (notably, the DM PI’s) characteristics early in the research process—considering their spheres of influence, associated limitations, even individual factors—and with this understanding, set reasonable boundaries and expectations for the research. An example of such calibration was seen in the case of Colombia, where the DM PI’s knowledge of system actors (and her understanding of the potential to influence them) led her to focus deliberately on change within her locus of control. We noted the opposite was true in the case of Bolivia, where the link between the decision-maker’s professional authority and the research-informed solutions was not as direct, limiting her ability to act on the evidence. This supports the potential value of constructing research teams based upon a strategic understanding of their spheres of influence.

Our analysis also highlighted a number of individual-level factors that can affect the extent to which DM PIs can effectively assume the role of research use “champion” [44]. Future studies on the role of such factors in shaping research use strategies are needed; for instance, in-depth analyses that explicitly apply relevant psychological theories on individual behaviour change [45], to understand how DM PI characteristics/attitudes affect their ability to advance EIDM.

### Ongoing assessment of evolving context and stakeholders

Implementing any kind of evidence-informed change demands a strong understanding of the evolving context and relevant stakeholders. To some extent, all three EIR teams informally assessed stakeholders in their early research planning and also engaged critical stakeholders in interviews to gauge their perceptions about the problem studied and possible solutions. These efforts, enhanced by the DM PI insider role within the targeted programmes/policies, improved EIR teams’ understanding of various stakeholders’ stance toward the issue. Nonetheless, we posit that additional benefits could be reaped from more formal, systematic and iterative processes to identify key stakeholders and to gauge their receptivity/interest in the research and potential influence over needed policy changes [36–38, 46].

One of the fundamental assumptions behind the EIR model is the existence of adequate (programme) decision-making spaces into which research evidence can be fed and ultimately considered—i.e. those receptive and amenable to EIDM. Such considerations could inform the focus of the research to an appropriate level of decision-making, thereby increasing the likelihood that EIR teams can take direct action. Contextual or situational analyses can help DM PIs: identify appropriate structures or processes through which evidence can be weighed and used to inform viable solutions, remain attuned to the evolving sociopolitical environment, and scope opportunities to influence programme/policy. We caution against overlooking the need for more structured approaches to both situational/contextual or stakeholder analysis—these are needed not just at the outset of the research, but also *after* findings are known to “update” earlier analyses, particularly as a year of changes can easily pass between research conceptualization and finalization of findings.

### Complexity, uncertainty and adaptation

As with most evidence-to-action processes in health systems, EIR is a complex enterprise. Our cases support references to the multiplicity of factors that intervene in the post-research processes as well as the potential interplay across them, occurring in dynamic contexts, over different levels of the system and often implicating a range of actors [10, 40, 47, 48]). All of this yields high levels of uncertainty, particularly as many of these factors are less susceptible to change by EIR teams. However, as others have posited, even factors outside the control of study teams should be identified and assessed in order to anticipate and plan for effective strategies to manage risks and enhance enabling factors [49]. All this requires EIR teams to continually adapt their approach to the circumstances—ideally guided by the type of iterative assessment previously mentioned. The literature on IR has stressed the importance of adaptation during the research itself, with regard to study design and methodology—i.e. adjusting methods in accordance with emerging information needs and contextual factors [9]—as well as context-informed modifications to the implementation strategies under study [4, 10, 50]. This study shows that such adaptation is also critical in processes related to research dissemination, engagement with the evidence, and its application to decision-making. Our cases adapted their research use strategies to the evolving circumstances through, for example, refocusing message framing; targeting dissemination to new stakeholders or decision-making bodies; and, in one case, adjusting remedial measures based on observed effects. Nonetheless, even with the best of assessments, it is not always possible to predict the effect of a particular factor [45]—the ability of EIR teams to tolerate (and persevere in) the context of uncertainty is key.

We observed that in the post-research phase, the ability of DM PIs to assume responsibility for remedial action can attenuate the complexity involved, to the extent that this simplifies the range of strategies needed—e.g. engaging stakeholders with the evidence—or, at least, reduces their intensity. Herein lies a key advantage of this EIR approach. Furthermore, some of the key barriers to research use by decision-makers—e.g. access to evidence, relevance of findings, leadership’s willingness to implement change, decision-maker and researcher interaction [13]—can be circumvented through EIR.

## Strengths and limitations

This theory-driven study applied a working conceptual framework of EIR, which guided the consistent, systematic examination of our cases. The prospective design with three data collection points allowed for follow-up on developments over time, serving a “member-checking” [51] function on respondents’ previous assertions. Prolonged engagement enhanced rapport with EIR teams. Interviews with potential knowledge users external to the study team reduced reliance on self-reporting by study teams and allowed for triangulation of data by respondent category. Triangulation by data source (document review and interviews) further enriched our analysis. Peer debriefing was conducted periodically with senior researchers to discuss all stages of the research.

Commonly cited challenges to studying complex processes of research use and decision-making were relevant in this study [52–56]. At times it was difficult to establish direct linkages between individual strategies employed by each team and the key outcomes achieved and to discern relative primacy across the multiple influencing factors. Additionally, respondents (being “insiders”) may not always recognize routine processes as relevant to the outcomes of interest in our study, resulting in inadvertent reporting omissions. Study team respondents were likely inclined to frame their experience in a positive light (social desirability); however, the inclusion of diverse respondents and follow-up interviews helped mitigate this bias. Some desired changes may require a longer time frame than was feasible for this study (11–24 months post-research) [52]. Nonetheless, the limited advances observed from the second and third rounds of data collection suggest that we captured core effects. Lastly, the iPIER approach to EIR is only one of many; some findings may be specific to this decision-maker-led model with built-in TA.

We recognize that there are certain built-in biases and risks to this model of EIR which relies heavily on a small number of decision-makers. While no pitfalls were mentioned by our respondents, potential drawbacks associated with involvement of system stakeholders in research have been described in the literature [4, 28, 57], including potential for loss of objectivity in interpretation of evidence. The role of decision-makers proposed in this model should be considered in light of both strengths and potential limitations, for example by supporting processes for decision-maker reflexivity and establishing suitable support and/or accountability mechanisms (e.g. scientific advisory committee).

## Conclusion

In this study, we examined the strategies through which EIR teams advance the aim of evidence-informed programme/policy improvements in the later-phase processes of EIDM. We found that EIR led by well-positioned DM PIs can facilitate impactful research translation efforts at both policy and service delivery levels. Given the complex, context-sensitive and dynamic nature of the processes involved, diverse strategies were employed across cases, establishing different pathways of change. We distinguish between two broad pathways of change: (1) the DM PI directly acts on the evidence within their sphere of authority and (2) the DM PI must engage other pivotal system stakeholders to redress study findings. Therefore, a key determinant of the overarching change pathway and the specific strategies needed was the positionality of the DM PI vis-à-vis the needed changes.

While identification of a prescriptive set of strategies was neither possible nor appropriate due to the complexity involved, findings may guide EIR practitioners in planning and conducting fit-for-purpose and context-sensitive strategies to advance the use of evidence for programme improvement in the post-research phase. The lessons emanating from this study about post-research EIR strategies point to three critical areas of “good practice”: the importance of understanding DM PI authority, ongoing assessment of the evolving context and relevant stakeholders, and iterative adaptation to dynamic and, at times, uncertain circumstances.

EIR can contribute to bridging implementation gaps, cultivating a culture of evidence and building the “learning health systems” needed to improve health outcomes globally. This paper has contributed to understanding the nuances of EIR post-research processes in low-resource contexts—processes which one respondent described as involving “the most challenging work” for EIR teams. We encourage other efforts such as ours to help build the nascent empirical knowledge base about decision-maker-led EIR approaches so that EIR practice is itself evidence-informed.

## Supplementary Information

**Additional file 1: Annex S1.** Summary table of research findings and recommendations for each case.

**Additional file 2: Annex S2.** Practical considerations for EIR to support use of evidence for health programme improvement.

## Data Availability

The datasets generated during and/or analysed during the current study are not publicly available in order to maintain confidentiality of the data but are available from the corresponding author on reasonable request.

## Abbreviations

Co-PI: Co-principal investigator
DM PI: Decision-maker principal investigator
DR: Dominican Republic
EIDM: Evidence-informed decision-making
EIR: Embedded implementation research
FP: Family planning
iPIER: Improving Program Implementation through Embedded Research initiative
IR: Implementation research
LAC: Latin America and the Caribbean
LMIC: Low- and middle-income countries
MOH: Ministry of Health
MOPH: Ministry of Public Health
PAHO: Pan American Health Organization
SNS: National Health Services acronym in Spanish
TA: Technical assistance
