# Supplementary material for: Strategies to promote evidence use for health programme improvement: learning from the experiences of embedded implementation research teams in Latin America and the Caribbean
Source: Health Res Policy Syst. 2022 Apr 7;20:38. doi: 10.1186/s12961-022-00834-1 (PMC8991468; doi:10.1186/s12961-022-00834-1)
Supplement: Supplementary file 1 — Additional file 1: Annex S1. Summary table of research findings and recommendations for each case. [file 12961_2022_834_MOESM1_ESM.docx]

**HRPS-D-21-00331**

**Annex S1: Summary Table of Research Findings and Recommendations for each Case**

|  | **Bolivia** | **Colombia** | **Dominican Republic** |
| --- | --- | --- | --- |
| **Targeted program** | *Chispitas* micronutrient supplement (one intervention within the wider national micronutrient strategy) | *Por Ti Mujer* (PTM) cervical cancer program, focusing on the screening component | National Family Planning Program (FPP) within the MoPH Sexual and Reproductive Health Program, focusing on male contraception component |
| Research Findings | 1) Health worker level: lack of updated knowledge/ capacity about *Chispitas*  2) Beneficiary level: lack of acceptability of *Chispitas* product for practical and cultural/ behavioral reasons  Many findings suggest inadequate assessment of local/cultural acceptability of intervention (*Chispitas* product) or piloting to adapt the intervention prior to national roll out (~2007) | Barriers: disconnect between perspective of service providers and service users with regard to access; request among service users for more human-centered care and greater integration with other health programs; non-users mention cultural beliefs and previous negative experiences with health system as barriers | The findings reveal tacit knowledge about the FP program: there is clear absence of a gender lens within the FP program (while known anecdotally and through their experience as decision-makers, there was no documented evidence of this gap).  Also revealed interest among men in male contraception [demand] |
| Policy/Program Recommendations | No specific changes to intervention implementation; focus on sensitizing mothers about the importance of the *Chispitas* to reduce anemia and also demonstrations on how they should be used (demand side focused)  Focus on: 1) health worker capacity development 2) carry out demonstrations of *Chispitas* preparation for mothers; on the whole, recommendations are very broad, not clearly actionable. | Focus on health work force capacity to improve service quality; strategizing among the administrators and managers to improve coverage; health information system strengthening and improved monitoring/analysis of relevant indicators; need for additional research on quality of services to be organized by health network managers.  Developed by iPIER research team, without external consultation, as focus was internal to the Health Network under study. | Recommendations for multiple strategies to communicate/educate about, build capacity for service delivery, create strategic alliances, and establish norms for male contraception  Recommendations largely drawn from the responses of the decision-makers and health professionals in study interviews  Additional recommendations emerged during the action planning workshop post-dissemination meeting |
