# Supplementary material for: Strategies to promote evidence use for health programme improvement: learning from the experiences of embedded implementation research teams in Latin America and the Caribbean
Source: Health Res Policy Syst. 2022 Apr 7;20:38. doi: 10.1186/s12961-022-00834-1 (PMC8991468; doi:10.1186/s12961-022-00834-1)
Supplement: Supplementary file 2 — Additional file 2: Annex S2. Practical considerations for EIR to support use of evidence for health programme improvement. [file 12961_2022_834_MOESM2_ESM.docx]

**HRPS-D-21-00331**

**Annex S2: Practical Considerations for EIR to Support Use of Evidence for Health Program Improvement**

| **For EIR Practitioners** |
| --- |
| *Rather than apply a pre-determined set of strategies to promote the use of research, EIR practitioners are encouraged to consider the factors and conditions in their own context that may shape the relevance and appropriateness of different strategies. Key to this planning phase are the following considerations:*   - Late-stage processes of evidence-informed decision-making need to be considered and planned from the beginning.   - Develop (and adapt) a research dissemination and utilization plan based on stakeholder analysis, to guide stakeholder engagement throughout the evidence-to-action processes   - Plan/budget for processes needed to interpret and translate findings into actionable recommendations (e.g. Consultative workshops, development of targeted communication/dissemination materials) at the outset of the research endeavor, adapting as circumstances evolve   - Identify strategies to integrate the research evidence within existing (program/policy) processes that can support its eventual use (e.g., quality improvement mechanisms, program reviews/evaluations, or relevant decision-making processes such as annual planning and budgeting); as possible, align study timing to ensure evidence can feed into these existing processes   - Establish Scientific Advisory Committee whose membership includes practitioners and higher-level decision-makers who can also facilitate learning, guide practice, and help bring continuity to the evidence-to-action processes, even in the context of decision-maker turnover - Tailor scope of evidence dissemination strategies based on the nature of the desired change and how this relates to 1) DM PI sphere of authority and 2) the breadth of stakeholders implicated in requisite change processes - Leverage stakeholder analysis to adapt message framing about key findings to different target audiences, determine suitable knowledge products, and need for more extensive deliberative processes - Determine at outset of research whether appropriate processes/structures exist through which to engage key stakeholders with evidence and ensure evidence is fed into decision-making; establish new decision-making structures or spaces (formal or informal), as relevant - Leverage decision-maker routine responsibilities as opportunities to integrate key findings into policy/practice - Engage in reflective practice to ensure ongoing critical monitoring of potential biases, particularly with respect to the implications of findings for action |
| **For Health Research Funders** |
| *Considerations to ensure that grant mechanisms allocate available resources to cases best situated to advance evidence-informed decision-making goals*  **Grant Mechanism Design—Requirements and Supports**   - Establish clearly defined criteria for selection of grant recipients to ensure:   - Suitable positionality of the decision-maker PI vis-à-vis the targeted research problem (i.e., authority and influence needed to engage directly in the use of findings)   - Decision-making processes/structures exists into which research processes can be integrated (i.e., where evidence can formally be reviewed and considered); promote creation of relevant structures, where lacking   - Wider stakeholder buy-in: Meaningful endorsement of the need for/relevance of the research by influential system stakeholders who can engage directly to support remedial actions emerging from the evidence - Incorporate structured stakeholder analysis as priority step early in the research to ensure that EIR teams identify the most critical stakeholders implicated by the research, understand their interests and priorities, and can engage them with the evidence in timely manner - Focus mentoring and capacity building for EIR teams not only on methodological/ research capacity, but also on other competencies needed for evidence-to-action processes (e.g., understanding how to identify implementation problems amenable to research, stakeholder engagement, knowledge and skills concerning partnership management into the post-research phase, strategizing for dissemination of findings, facilitating deliberative dialogue and consultative processes, and other facets of research translation). - Engage high-level MOH decision-makers (e.g. Policy, Planning and Budgeting units) in research grant stewardship and oversight, including facilitation and support to research utilization strategies   **Institutionalization of IR within Health Systems**   - Support MoH to create an enabling ecosystem for use of research in decision-making   - Establish (or mandate) processes for integrating research into decision-making in program/policy cycles   - Create organizational and individual incentives for decision-makers to use evidence as well as for researchers to engage in EIR alongside decision-makers (and accept a position of “power-sharing” with DMs);   - Establish institutional strategies to link decision-makers with researchers (e.g., through formal communities of practice or other professional networks);   - Set up accountability systems/structures to ensure these initiatives are implemented with rigor and that evidence is deliberately considered in decision-making   **Funding**   - Ensure EIR initiatives also include funding for rigorous, prospective evaluation of projects that extends well beyond the research endeavor into the post-research evidence-to-action phase |
